# Supplementary material for: Embodied intelligence via learning and evolution
Source: Nat Commun. 2021 Oct 6;12:5721. doi: 10.1038/s41467-021-25874-z (PMC8494941; doi:10.1038/s41467-021-25874-z)
Supplement: Supplementary file 1 — Supplementary Information [file 41467_2021_25874_MOESM1_ESM.pdf]

Embodied Intelligence via Learning and Evolution

Supplementary Information

1 Supplementary Figures

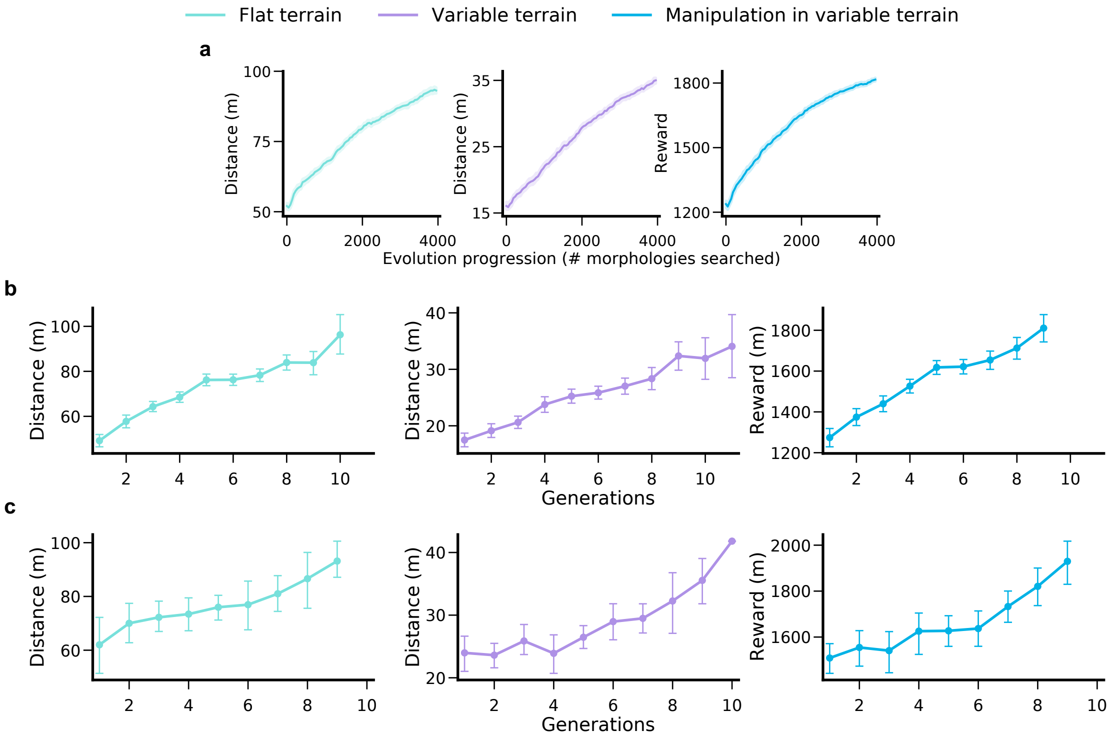

Supplementary Figure 1: **Role of evolution in fitness enhancement. a** Progression of the mean ($n = 300$) fitness of the top 100 agents in each of 3 evolutionary runs. **b** Progression of mean ($n = 100$) fitness for the lineages of the best 100 agents (same as Fig. 4a,c) in the final population across 3 evolutionary runs. The fitness of the agents towards the end of evolution is almost double that of their early ancestors. **c** We select the 10 best agents, out of 576 initial agents at generation 0, in each evolutionary run for each environment, whose lineages survive till the end of evolution. We then plot the mean ($n = 30$) fitness of these agents across generations following the lineage of its best descendant in the final population. The performance, even for the 10 best initial agents, increases over evolution by a factor of 1.5 to 2 across three environments. Shaded region in **a** and error bars in **b**,**c** denote 95% bootstrapped confidence interval.

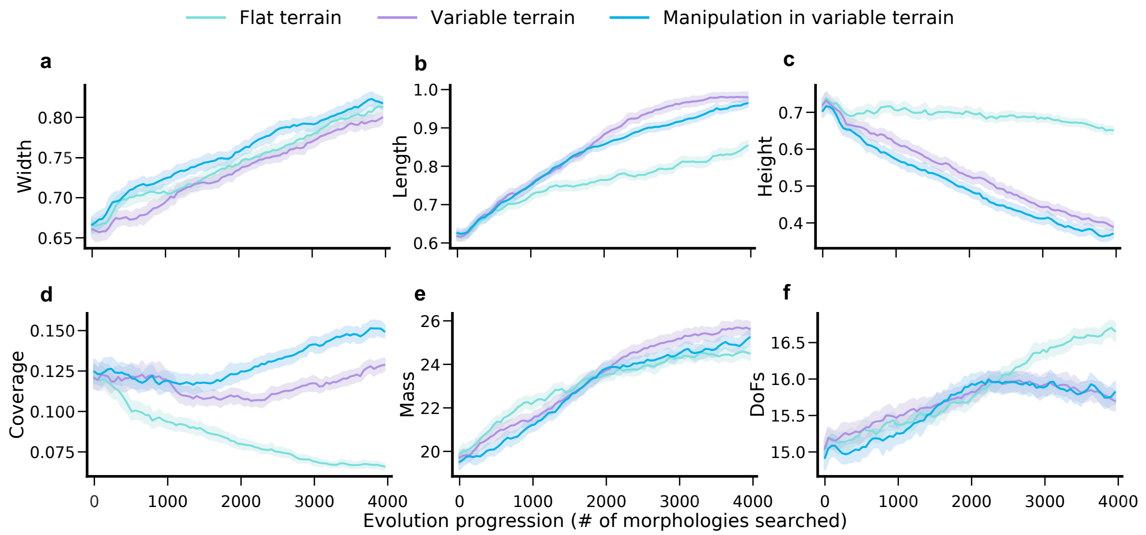

Supplementary Figure 2: **Influence of environment on different morphological descriptors.** (**a-e**) Progression of the mean of different morphological descriptors averaged over 3 runs in different environments for the entire population. Shaded region denotes 95% bootstrapped confidence interval. (**a-c**) VT/MVT agents tend to be longer along the direction of forward motion and shorter in height compared to agents evolved in FT. **d** Coverage is the ratio of volume of the agent morphology and it's axis aligned bounding box. FT agents are less space filling as compared to VT and MVT agents. Agents evolved in all three environments have the similar masses (**e**) and DoFs (**f**).

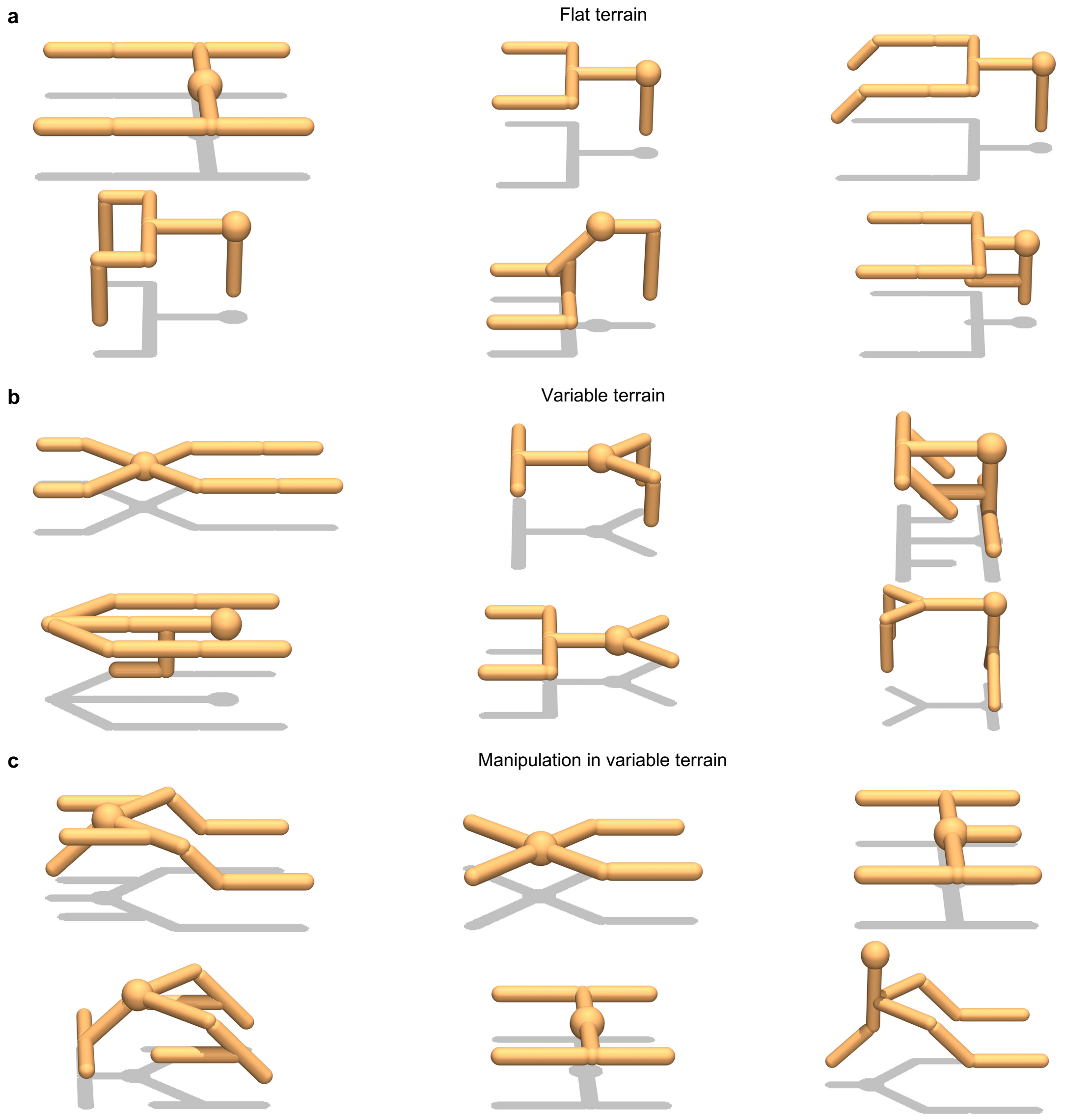

Supplementary Figure 3: **Best agent morphologies evolved in different environments.** A subset of the top 10 agent morphologies evolved across 3 evolutionary runs. See evaluation methodology in Methods for details about selection procedure and Supplementary Video for illustration of learnt behaviour.

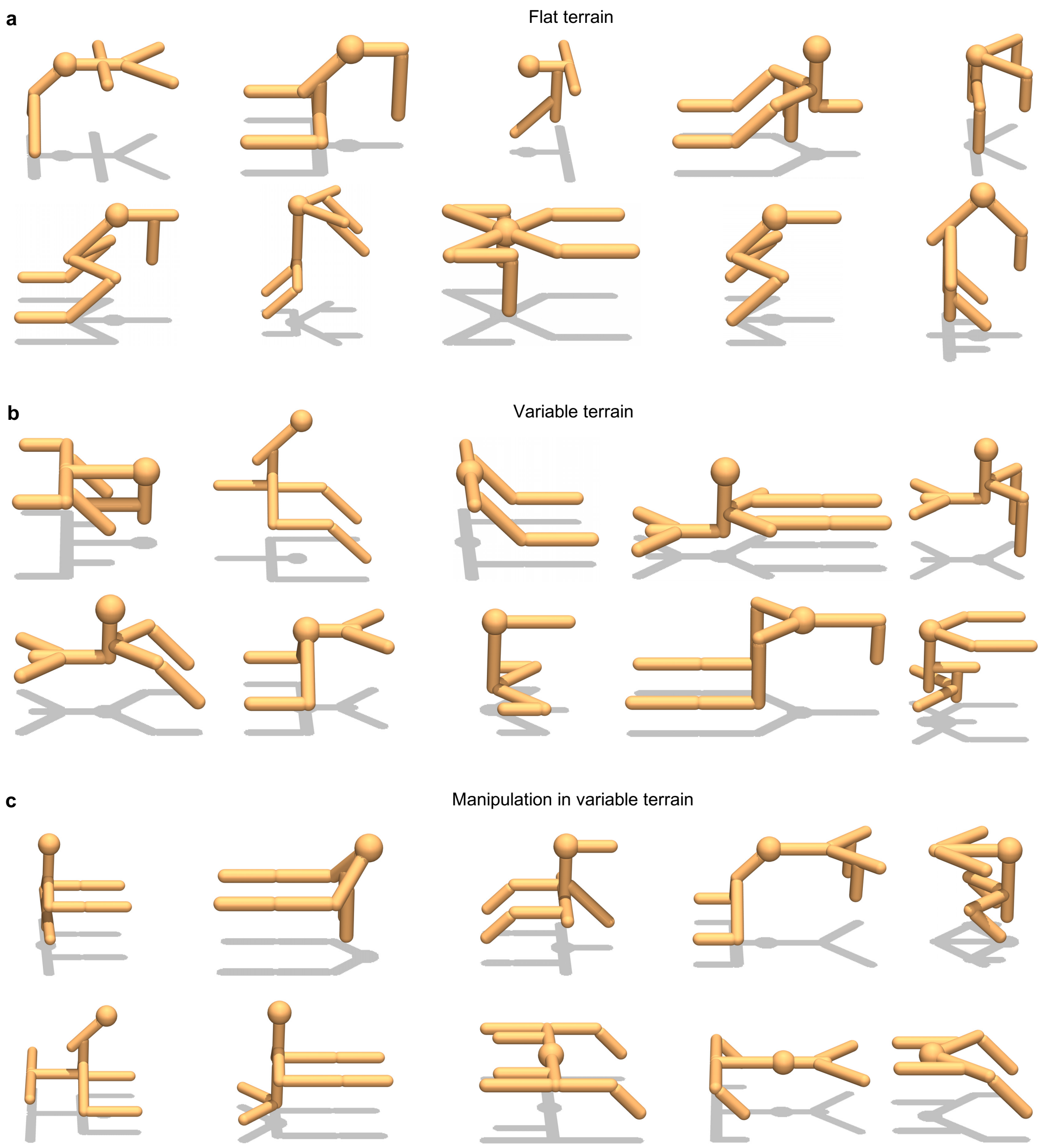

Supplementary Figure 4: **Example agent morphologies evolved in different environments.** A subset of the top 100 agent morphologies evolved across 3 evolutionary runs. See evaluation methodology in Methods for details about selection procedure.

2 Supplementary Tables

Hyperparameter	Value
Max limbs	10
Limb radius	0.05
Limb height	$[0.2, 0.3, 0.4]$
Limb density	$[500, 600, 700, 800, 900, 1000]$
Limb orientation theta	$[0, 45, 90, 135, 180, 225, 270, 315]$
Limb orientation phi	$[90, 135, 180]$
Head radius	0.10
Head density	$[500, 600, 700, 800, 900, 1000]$
Joint axis	$[x, y, xy]$
Motor gear range	$[150, 200, 250, 300]$
Joint limits	$[(-30, 0), (0, 30), (-30, 30),$ $(-45, 45), (-45, 0), (0, 45),$ $(-60, 0), (0, 60), (-60, 60)$ $(-90, 0), (0, 90), (-60, 30)(-30, 60)]$

Supplementary Table 1: **Hyperparameters for UNIMAL design space.** Mutation operations choose a random element from the corresponding list of possible parameters. The set of all possible values of these hyperparameter choices yields an estimate of 10^{18} possible morphologies.

**Hyperparameter**	**Value**
Discount γ	.99
GAE parameter λ	0.95
PPO clipping parameter ϵ	0.2
Policy epochs	4
Batch size	512
Entropy coefficient	0.01
Reward normalization	Yes
Reward clipping	$[-10, 10]$
Observation normalization	Yes
Observation clipping	$[-10, 10]$
Timesteps per rollout	128
# Workers	4
# Environments	32
Total timesteps	5×10^6
Optimizer	Adam
Initial learning rate	0.0003
Learning rate schedule	Linear decay
Gradient clipping (l_2 norm)	0.5
Clipped value function	Yes
Value loss coefficient	0.5

Supplementary Table 2: **PPO hyperparameters.**
