# Supplementary material for: Embodied intelligence via learning and evolution
Source: Nat Commun. 2021 Oct 6;12:5721. doi: 10.1038/s41467-021-25874-z (PMC8494941; doi:10.1038/s41467-021-25874-z)
Supplement: Supplementary file 2 — Description of Additional Supplementary Files [file 41467_2021_25874_MOESM2_ESM.docx]

Description of Additional Supplementary Files

Title: Supplementary Movie 1

Description: This video shows agents evolved in different evolutionary environments and performing different evaluation tasks.
